# Factors Associated with the Perception of Obstetric Violence and Its Emotional Impact on Healthcare Training: A Cross-Sectional Study

**DOI:** 10.3390/nursrep15120425

**Published:** 2025-11-28

**Authors:** Irene Llagostera-Reverter, Víctor Ortíz-Mallasén, Marisol Mejuto-Prego, Desirée Mena-Tudela

**Affiliations:** 1 Nursing Research Group (GIENF Code 241)-Nursing Department, Universitat Jaume I, Avinguda de Vicent Sos Baynat, s/n, 12071 Castellón de la Plana, Comunidad Valenciana, Spain; llagoste@uji.es (I.L.-R.); dmena@uji.es (D.M.-T.); 2 School of Nursing, Universidade da Coruña, As Xubias, 84, 15006 A Coruña, Galicia, Spain; al441630@uji.es; 3 Centro de Salud de Vimianzo-Área Sanitaria da Coruña e Cee, Rúa Rosalía de Castro, s/n, 15129 A Coruña, Galicia, Spain; 4 Feminist Institute, Universitat Jaume I, Avinguda de Vicent Sos Baynat, s/n, 12071 Castellón de la Plana, Comunidad Valenciana, Spain

**Keywords:** obstetric violence, health professional education, feminist perspective, reproductive rights, nursing students, respectful maternity care

## Abstract

**Background/Objectives:** Obstetric violence (OV) is a violation of women’s human rights during reproductive processes. Despite being the subject of debate among healthcare professionals, increasingly recognized, and legislated against in some countries, OV continues to be reproduced and normalized during training. The objective of this study was to determine the perception of OV among health sciences students and gynaecology and obstetrics residents. **Methods**: A cross-sectional observational study was conducted with 304 health sciences students and gynaecology and obstetrics residents in Spain. An online questionnaire was distributed that gathered information on sociodemographic variables and clinical experience and included the validated PercOV-S instrument. Descriptive and bivariate analyses were performed to explore associations between variables. **Results**: The overall perception of OV was moderately high (mean 3.93/5), with higher scores for visible or protocolized forms (4.27/5) than for invisible or subtle forms (2.87/5). Being a woman, being a midwifery resident, or having had personal experiences with pregnancy or childbirth increased sensitivity to OV. Clinical exposure in obstetrics and gynaecology services increased both awareness and the likelihood of witnessing OV. Twenty-eight percent of students reported having observed OV, and twenty percent reported emotional distress, even considering dropping out. **Conclusions**: Despite the recognition of OV, repeated exposure during training can promote its normalization. The results of this study highlight the need for safe, reflective training environments that mainstream feminist perspectives, sexual rights, and the detection of subtle forms of OV.

## 1. Introduction

The World Health Organization expressed its concern about the mistreatment that existed during childbirth a few years ago [1]. Obstetric violence (OV) is a form of gender-based violence perpetrated against women during obstetric care [2]. Although the concept has gained visibility in recent decades, its roots lie in the progressive institutionalization of childbirth care, which displaced women from the sphere of caregiving. The interrelation between patriarchy, biomedicine, and institutional structures often fails to prioritize women’s autonomy, contributing to the medicalization of physiological processes and the denial of informed consent [3]. Likewise, obstetric violence is defined as the appropriation, by healthcare personnel, of women’s bodies and reproductive processes, expressed through dehumanizing treatment, excessive medicalization, and loss of autonomy [2]. It also entails ignoring women’s needs, desires, and dignity during gynaecological and obstetric care [4]. Thus, two central axes are identified in this type of violence: the loss of women’s autonomy and the resulting physical, emotional, psychological, and socio-familial harm [5,6,7,8]. Spain has a significant public health problem with obstetric violence [7]. Various studies show that obstetric violence rates in Spain range from 38% to 67% [7,9].

However, women are not the only ones affected. Kendall-Tackett et al. [10] emphasize that maternal care professionals—such as nurses, midwives, and obstetricians—may experience secondary traumatic stress and moral injury as a result of exposure to traumatic births and obstetric violence, which can negatively impact their emotional well-being and professional performance.

One of the most concerning aspects of obstetric violence is that it continues to be ignored—or even denied—by large sectors of the medical community, despite efforts toward recognition and mitigation. In Spain, the Spanish Society of Gynaecology and Obstetrics has publicly denied the existence of this type of violence, labelling the term “scientifically unacceptable” [11]. Such denialism contributes to the social normalization of abusive practices, hindering their identification by both the general public and healthcare users. The symbolic authority of healthcare professionals reinforces this invisibility and legitimizes violent practices under the guise of normality [12]. Academic training plays a key role in this normalization process [13]. Although students recognize the presence of obstetric violence in their training environments, frequent exposure to these practices by their superiors may lead to normalization [14]. Evidence shows that while future healthcare professionals clearly identify obstetric violence during their early years of training, this perception tends to diminish as they progress in their studies, suggesting a process of progressive naturalization [15]. This underscores the urgent need to investigate the mechanisms through which obstetric violence is normalized within health education and to implement effective educational strategies to counteract it [15]. The perception of obstetric violence among students and healthcare professionals is, therefore, a critical indicator for understanding the degree of internalization of these practices and the effectiveness of educational programs in integrating feminist and human rights perspectives [16].

Furthermore, training interventions focused on obstetric violence—whether in-person or online—have been shown to significantly improve students’ awareness and engagement and can be effectively adapted to different contexts and methodologies [15,17].

The aim of this study is to determine the level of perception of obstetric violence and appropriate gynaeco-obstetric interventions among health sciences students and residents in Spain. Through analysis of these perceptions, the study contributes to critical reflection on the explicit and hidden curriculum in health education and promotes pedagogical strategies that foster a respectful, woman-centred, evidence-based model of care grounded in human rights.

## 2. Materials and Methods

### 2.1. Study Design and Participants

A cross-sectional observational study was conducted to determine the level of perception among health sciences students regarding obstetric violence and appropriate gynaecological and obstetric interventions.

The study population comprised undergraduate students from health sciences disciplines (medicine, nursing, psychology, and others), as well as medical and nursing residents (MIRs and EIRs, respectively) specializing in obstetrics and gynaecology in Spain. Thus, the inclusion criteria taken into account were: undergraduate students in health sciences disciplines (medicine, nursing, psychology, and others), as well as residents (MIR and EIR) in obstetrics and gynaecology in Spain. Questionnaires with more than 10% of missing responses were excluded.

Sample size calculation was performed using GRANMO software version 7.12 (Barcelona, Spain), which indicated that a sample of 236 participants was sufficient to estimate, with a 95% confidence level, a population proportion of 60% with a precision of ±7 percentage points. A potential loss rate of 20% was considered.

A total of 305 responses were obtained, among which one survey (0.32%) was excluded because the participant left more than 10% of the questions unanswered. The final sample consisted of 304 health sciences students.

### 2.2. Variables

This study included ad hoc sociodemographic variables, obstetric data, childbirth experience, treatment during obstetric care, type of discrimination, education in health and gender, management of obstetric violence, exposure to obstetric violence during training, and type of violence witnessed during education.

In addition, students’ perception of obstetric violence was measured using the PercOV-S instrument, which consists of 33 items rated on a 5-point Likert scale. PercOV-S was an instrument developed and validated in Spanish health sciences students, where 1 indicates the lowest level of perception/agreement and 5 the highest. It has demonstrated strong internal consistency for both the total scale (α = 0.936) and its dimensions: visible–protocolized obstetric violence (α = 0.802) and invisible–non-protocolized obstetric violence (α = 0.952) [16]. In this study, the PercOV-S showed high internal consistency overall (α = 0.930) and for both dimensions: visible–protocolized (α = 0.847) and invisible–non-protocolized obstetric violence (α = 0.906).

### 2.3. Data Collection

Data were collected through a national online survey distributed via institutional email and social media between April and June 2023. The first question of the survey requested that the participants confirm their consent to take part in this study.

### 2.4. Data Analysis

A descriptive analysis was performed using means, standard deviations, and 95% confidence intervals for quantitative variables. For qualitative variables, frequency distributions and percentages were calculated.

A descriptive analysis of the PercOV-S instrument was carried out to identify which items were easier or more difficult for the participants to interpret. Since the data distribution did not meet the assumption of normality (*p* < 0.001 in the Kolmogorov–Smirnov test), bivariate analyses were conducted using non-parametric tests (Mann–Whitney U and Kruskal–Wallis tests) to identify possible associations between study variables and PercOV-S scores, both globally and by dimension. The χ^2^ test or Fisher’s exact test was used for categorical variables.

Statistical analyses were performed using SPSS version 25 and Jamovi version 2.3.28. Graphs were created using Microsoft Excel spreadsheets. A significance level of *p* < 0.05 was established for hypothesis testing.

### 2.5. Ethical Considerations

This study was conducted in accordance with Regulation (EU) 2016/679 of the European Parliament and of the Council of 7 April 2016 on the protection of natural persons, the Spanish Organic Law 3/2018 of 5 December on Personal Data Protection and Digital Rights Guarantee, and the principles of the Declaration of Helsinki. This study was approved by the Human Research Ethics Committee of the Universitat Jaume I (CD/26/2020).

## 3. Results

### 3.1. Descriptive Analysis

The mean age of the sample was 22.95 years (±5.481). The majority were women (88.9%, *n* = 273). Most participants were born in Spain (94.4%, *n* = 287).

Regarding academic background, 53.0% (*n* = 161) were nursing students, 15.5% (*n* = 47) studied medicine, 14.1% (*n* = 42) studied psychology, 12.2% (*n* = 37) were midwifery residents, and 5.3% (*n* = 16) were enrolled in other health-related disciplines (pharmacy, physiotherapy, and dentistry). In terms of academic year, most students were in their second year (28.3%, *n* = 86).

With respect to obstetric variables, 10.5% (*n* = 32) of participants reported having experienced at least one pregnancy, and among these, 62.8% (*n* = 22) had given birth. Additionally, 19.7% (*n* = 60) indicated having personally experienced some form of obstetric violence.

Regarding childbirth experience, 37.2% (*n* = 113) reported having witnessed at least one childbirth, and 41.4% (*n* = 126) stated that they had worked or completed clinical placements in gynaecology and obstetrics. Among these, 68.3% (*n* = 86) reported that consent was requested from the woman before entering the room or delivery area for obstetric care.

**Figure 1:**
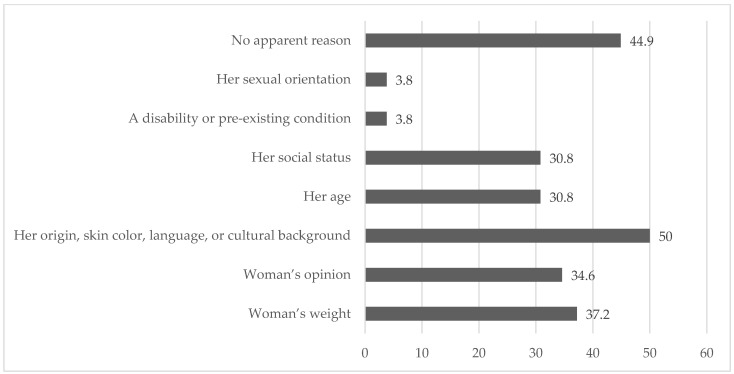
Observed percentage of types of discriminatory treatment experienced by women receiving obstetric care.

Concerning equity in the treatment of women during obstetric care, 61.9% (*n* = 78) stated that they had observed differences in how women were treated. The most frequently perceived reasons for these differences were “origin, skin colour, language, or cultural background” (50%, *n* = 39), “no apparent reason” (44.9%, *n* = 35), and “the woman’s weight” (37.2%, *n* = 29) (Figure 1).

Regarding training in health and gender, 76.3% (*n* = 232) of respondents reported having received courses or subjects that incorporated a feminist perspective during their academic education. Additionally, 78.6% (*n* = 239) stated that they had previously heard of the concept of obstetric violence.

With respect to knowledge of legislative initiatives related to obstetric violence, 32.9% (*n* = 100) of the sample indicated being aware that Spain had expressed an intention to legislate on the matter. On the other hand, most participants (88.5%, *n* = 269) agreed with the use of the term “obstetric violence”, and 71.1% (*n* = 216) believed that this concept does not criminalize healthcare professionals.

During their academic training, 28.0% (*n* = 85) of participants reported having witnessed at least one event of obstetric violence. The most frequently observed examples were a “lack of privacy for women” (83.5%, *n* = 71), “derogatory comments toward women” (70.6%, *n* = 60), and “insufficient time devoted to their care” (61.2%, *n* = 52). Exposure to this type of violence caused anxiety (55.6%, *n* = 48), hypervigilance (50.6%, *n* = 43), and agitation (38.8%, *n* = 33) in students. A proportion of students (20.0%, *n* = 17) reported having considered temporarily withdrawing from their studies (Table 1).

### 3.2. Overall Perception of Obstetric Violence

The overall perception of obstetric violence, assessed using the PercOV-S instrument, showed a mean global score of 3.93 (±0.593), indicating a moderately high perception among the students. When analysing the two differentiated dimensions of the instrument, the protocolized–visible obstetric violence dimension obtained the highest mean score (4.27 ± 0.539), compared to the non-protocolized–invisible obstetric violence dimension (2.87 ± 0.921).

### 3.3. Relationship Between Collected Variables and PercOV-S Instrument

Table 2 presents the descriptive data and the differences observed in the PercOV-S scores, both globally and by dimensions. The global score of the instrument showed statistically significant differences according to sex and field of study (*p* < 0.001), previous pregnancy (*p* = 0.006), previous childbirth (*p* = 0.014), having personally experienced a situation of obstetric violence, having witnessed a childbirth, having worked or completed clinical training in the field of gynaecology and obstetrics, and perceiving equal treatment (*p* < 0.001). Regarding the type of discrimination observed, significant differences were found for women’s weight (*p* = 0.005), women’s opinion (*p* = 0.038), and age (*p* = 0.049). Significant associations were also identified with having previously heard about the concept of obstetric violence, agreeing with the term, believing that it criminalizes healthcare professionals, and having witnessed a situation of obstetric violence (*p* < 0.001).

With respect to the types of obstetric violence witnessed, significant differences were found for interventions without informed consent (*p* = 0.003), telling women they were endangering their baby’s life or health (*p* = 0.030), physical violence (*p* = 0.001), and use of the Kristeller manoeuvre to accelerate labour (*p* = 0.041). Students who witnessed these practices reported emotional reactions such as trouble sleeping (*p* = 0.007), agitation (*p* = 0.014), and other unlisted feelings (*p* = 0.045), all statistically significant.

The protocolized–visible obstetric violence dimension showed statistically significant differences according to sex (*p* = 0.005), field of study (*p* < 0.001), previous pregnancy (*p* = 0.011), previous childbirth (*p* = 0.017), having personally experienced obstetric violence, having witnessed a childbirth, having worked or completed clinical training in the field of gynaecology and obstetrics, and perceiving equal treatment (*p* < 0.001). Regarding the type of discrimination, only women’s weight was significantly associated (*p* = 0.009). Significant associations were also observed for having heard about the concept of obstetric violence (*p* < 0.001), agreeing with the term (*p* = 0.003), believing that it criminalizes professionals, and having witnessed a situation of obstetric violence (*p* < 0.001). Among the types of obstetric violence observed during training, significant differences were found for interventions without informed consent (*p* = 0.001), physical violence (*p* = 0.015), and denial of desired treatments (*p* = 0.033). Witnessing these situations was significantly associated with trouble sleeping (*p* = 0.002) and agitation (*p* = 0.014).

The non-protocolized–invisible obstetric violence dimension also showed statistically significant differences according to sex and field of study (*p* < 0.001), previous pregnancy (*p* = 0.010), previous childbirth (*p* = 0.026), having personally experienced obstetric violence, having worked or completed clinical training in gynaecology and obstetrics, and perceiving equal treatment (*p* < 0.001). Regarding the type of discrimination observed, significant associations were found for women’s weight (*p* = 0.009) and women’s opinion (*p* = 0.032). Statistically significant relationships were also identified for having heard about the concept of obstetric violence, agreeing with the term, believing that it criminalizes professionals, and having witnessed a situation of obstetric violence (*p* < 0.001). Among the types of obstetric violence witnessed, significant associations were found for interventions without informed consent (*p* = 0.020) and physical violence (*p* < 0.001). Students reported significant associations between witnessing these events and trouble sleeping (*p* = 0.031), agitation (*p* = 0.048), and none of the above (*p* = 0.040).

Supplementary Material S1 shows the descriptive results and the differences observed for the overall score and by dimensions of the PercOV-S with the rest of the variables included in the study that were not statistically significant (*p* > 0.05).

### 3.4. Relationship Between Sex and Academic Background and the Rest of the Variables Included in the Study

Regarding sex and academic background, both showed significant differences for the following variables: agreeing with the term (*p* = 0.001; *p* = 0.022), believing that the term criminalizes health professionals (*p* = 0.035; *p* < 0.001), having witnessed equal treatment of women in obstetric care (*p* = 0.001), and having witnessed an OV situation (*p* = 0.016; *p* < 0.001). In relation to the type of OV observed during training, only the variable environment with physical violence (*p* = 0.013) and the variables interventions without CI, Kristeller manoeuvre, and having denied the women treatment or encouraged them to accept an intervention they did not want (*p* < 0.001) showed significant associations (Table 3).

Supplementary Material S2 provides the results and comparisons of the remaining sociodemographic variables with the other variables included in the study, which were not statistically significant for the most part but are important to consider.

## 4. Discussion

This study’s descriptive analysis provides a detailed portrait of the sociodemographic profile, clinical experience, and level of awareness of obstetric violence among medical and nursing students and residents specializing in obstetrics and gynaecology (EIRs and MIRs) in Spain. These findings reinforce the need to incorporate structured content on sexual and reproductive rights, ethics of care, and respectful maternity care into curricula to halt the perpetuation of obstetric violence in the earliest stages of professional education [18].

The predominance of women in the sample reflects the well-documented trend toward the feminization of healthcare professions, particularly nursing, where women have historically been the majority [19]. Beyond being a structural feature of the sector, this phenomenon has implications for how care practices are perceived and recognized, as women tend to display greater receptivity in identifying situations of disrespect or violations of rights during obstetric care [20]. This heightened sensitivity may be related both to gender socialization and to personal or collective experiences surrounding reproductive health. Bivariate analysis also confirms that sex was one of the most influential factors: women reported significantly higher scores than men in both the global and dimensional measures of obstetric violence perception. This finding is consistent with previous research indicating that gender socialization [19] and prior reproductive health experiences increase empathy and the ability to identify disrespectful or abusive practices in obstetric care [21]. Direct or indirect exposure to physical and emotional vulnerability during pregnancy and childbirth enhances critical awareness and rejection of violence—patterns that are more prevalent among women than men [20].

Another noteworthy aspect concerns the participants’ country of origin: those not born in Spain showed lower sensitivity in perceiving obstetric violence. Although the subgroup was small and results did not reach statistical significance, this finding is relevant since comparative studies have shown that professionals from contexts where certain practices are normalized—such as India, in comparison to the United Kingdom—are less likely to identify them as forms of violence [22]. This cultural difference points to a methodological limitation of the present study but also a promising line of future research: analysing, from an international perspective, how sociocultural contexts shape the perception and recognition of obstetric violence.

Regarding clinical exposure, the fact that many students had rotated through obstetrics and gynecology services and/or witnessed childbirth indicates that a substantial proportion had direct contact with obstetric care settings, which are key contexts for professional learning. The literature highlights that such clinical experiences are moments in which models of care are internalized—either perpetuating respectful practices or reinforcing normalized patterns of abuse or mistreatment [23]. The bivariate analysis also showed an effect on clinical practice exposure. Thus, having completed rotations in obstetrics and gynecology was associated with higher scores in both global perception and the visible-protocolized dimension. This suggests that direct exposure to childbirth care settings increases the likelihood of witnessing obstetric violence. However, this experience may have a dual effect: on the one hand, it allows harmful practices to be identified, but on the other, without adequate supervision and reflection, it may contribute to their normalization through vicarious learning [14].

The relationship between perception and direct exposure to specific practices was particularly significant: interventions performed without informed consent (e.g., medication administration, episiotomies, caesarean sections), physical violence (e.g., aggressive manipulations without anesthesia or repeated vaginal examinations), and the use of the Kristeller manoeuvre to accelerate labour were all clearly associated with higher scores in both visible and invisible dimensions. This highlights how direct observation of ethically questionable or abusive practices fosters a more critical professional awareness, emphasizing the formative impact of such experiences [24]. Despite this, institutional hierarchies position healthcare professionals as central figures of authority, relegating students to the lowest rung and preventing horizontal relationships during care processes [18]. This dynamic limits students’ ability to challenge inappropriate practices [13]. Thus, although students may play a crucial role in supporting women during childbirth and reporting mistreatment, their lack of specific training, absence of clear guidelines, and fear of reprisal hinder such actions [25].

That more than one-quarter of the sample reported having witnessed obstetric violence during their training suggests that these episodes may not be exceptional. This finding aligns with Bohren et al.’s review [26], which identified verbal abuse and lack of consent as the most common manifestations of OV across multiple contexts. The high percentage of observed discrimination is particularly concerning and consistent with studies describing how obstetric violence intersects with other forms of inequality and stigmatization, creating an intersectional scenario of rights violations [27]. The bivariate analysis also emphasized the relevance of witnessing discriminatory treatment during obstetric care—particularly discrimination based on body weight, age, or women’s opinions—which was associated with higher perception scores across dimensions. This reinforces the need to approach obstetric violence from an intersectional perspective that addresses multiple forms of exclusion and stigma, as these factors exacerbate vulnerability and perpetuate inequality in care [7,28].

A large proportion of participants reported having previously heard about obstetric violence and agreed with the use of the term. Compared with other contexts in which familiarity with the concept remains limited, this suggests that in Spain, obstetric violence has gained ground as a social and academic concept [20]. Political and legislative debate, media coverage, and feminist activism campaigns may have contributed to this increased recognition. However, being familiar with the term does not necessarily imply identifying all its manifestations, especially the subtler ones—underscoring the need for further research and the need to strengthen training, in line with the results obtained. Additionally, the PercOV-S score patterns confirm the trend observed in previous studies: the more visible, institutionalized, and protocolized forms of obstetric violence are, the easier they are to recognize, while “invisible” violence—expressed through gestures, omissions, or paternalistic attitudes—requires specific training to detect [7]. This finding is critical, since subtle forms of violence have a high potential to perpetuate hostile care environments without being sanctioned.

The bivariate analysis conducted in this study identified several sociodemographic, educational, and experiential factors significantly associated with the perception of obstetric violence according to the different PercOV-S dimensions, providing a more nuanced understanding of the determinants influencing students’ and residents’ sensitivity.

The field of study emerged as a key determinant: midwifery residents and nursing students obtained the highest perception scores, surpassing those of participants from psychology, medicine, and other programs. This pattern may reflect the fact that disciplines oriented toward holistic and woman-centred care incorporate curricula related to respectful childbirth, reproductive rights, medical ethics, and empathetic communication more intensively [21]. Moreover, the higher frequency of direct contact with childbirth care in these disciplines facilitates the detection of abusive practices, whereas medical training may focus more on technical aspects than on ethical or humanistic dimensions [29]. Thus, it can also be understood that most observers belonged to the groups with the highest exposure (nurses and resident midwives) and that this clinical environment is precisely the breeding ground for vicarious learning and the normalization of these practices. This phenomenon can be explained through Bandura’s concept of vicarious learning [30], whereby individuals adopt behaviors observed in others—especially authority figures—without directly experiencing them. When such behaviors are justified by factors such as occupational stress or the personal characteristics of professionals, their implicit acceptance is reinforced, perpetuating their reproduction in clinical settings and fostering the transmission of harmful practices to new generations of professionals. Further research is needed in this field.

Personal experiences related to pregnancy or childbirth were also strongly associated with higher sensitivity to obstetric violence. Such lived experiences provide concrete reference frameworks for recognizing disrespect, negligence, or structural violence—a phenomenon supported by the literature, which highlights how reproductive experiences shape critical perceptions of medical interventions and clinical care [27]. This pattern may be linked to gender socialization factors and to women’s greater tendency to verbalize critical experiences and recognize inequality dynamics [4]. This does not necessarily indicate insensitivity among men but rather a greater difficulty in identifying or acknowledging such practices in their own behaviour—reinforcing the need for training interventions that promote self-reflection and ethical awareness among all students.

The emotional impact experienced by the students after witnessing obstetric violence deserves special attention, as it not only had short-term consequences but, in some cases, affected their academic commitment. Indeed, a notable proportion reported considering temporarily interrupting their studies after such experiences, underscoring the need for safe and emotionally sustainable learning environments. These findings are consistent with the Spanish Ministry of Health’s 2024 report [31]. The literature also associates such experiences with secondary traumatic stress, a phenomenon affecting professionals and students who witness patient mistreatment or rights violations, generating emotional and cognitive repercussions that may undermine motivation and future ethical engagement [29]. Finally, the emotional symptoms resulting from exposure to these situations—such as sleep disturbances, agitation, and hypervigilance—were also associated with greater perception of obstetric violence. This underscores the importance of considering the psychological impact that witnessing such practices has on students. These experiences can serve as a catalyst for ethical change or, if inadequately addressed, contribute to professional burnout and disengagement [32].

This study presents several limitations. Its descriptive and cross-sectional design prevents establishing causal relationships, and the use of self-reported data may have introduced response bias. While self-reporting is inherent in the measurement of vicarious trauma, it cannot establish causality or a formal clinical diagnosis, only the association between the observation of obstetric violence and the manifestation of these symptoms. It is important to note that, despite the adequate sample size, the heterogeneity of the sample may dilute the normalizing effect of obstetric violence. Future research would benefit from studies focused exclusively on professions with high clinical exposure. The predominance of women and the non-probabilistic nature of the sample limit the generalizability of the findings, especially across genders and cultural contexts. In addition, the small number of participants born outside Spain reduces the robustness of cross-cultural comparisons. Finally, as data were collected within a specific institutional and national context, the results should be interpreted with caution when applied to other settings. Despite these limitations, further research is needed to deepen understanding in this area, with the aim of improving the quality of learning and, ultimately, enhancing the quality of care provided to all women.

Future research should aim to assess academic engagement and motivation, exploring relationships between the perception of obstetric violence, emotional impact, and potential study interruptions. Stratified analyses with more balanced samples by sex and country of origin would also allow for a deeper understanding of how these factors interact with the perception of obstetric violence. Finally, cross-cultural comparisons represent a particularly promising avenue to examine how cultural frameworks and models of care influence sensitivity to these practices.

## 5. Conclusions

This study reveals a moderately high perception of obstetric violence among nursing students and midwifery residents in Spain, particularly regarding its visible-protocolized dimension. Women, midwifery residents, and those with personal experiences of pregnancy or childbirth showed greater sensitivity to these situations. Clinical exposure during obstetrics and gynaecology rotations increased both awareness and the likelihood of witnessing obstetric violence, generating a significant emotional impact on part of the student body—some of whom even considered temporarily abandoning their studies.

Conversely, frequent exposure to such practices by authority figures may be associated with their normalization through vicarious learning. We cannot overlook the findings related to the emotional impact, the manifestation of temporary abandonment of studies, or the factors of discrimination that were perceived as reasons for unequal treatment (origin, weight of the woman, opinion, and age) associated with the perception of obstetric violence. These findings highlight the need to promote safe, supervised, and ethically reflective learning environments that foster respectful, dignified, intersectional and autonomous care for women.

Overall, the results underscore the importance of strengthening training in feminist perspectives, sexual and reproductive rights, appropriate gynaecological and obstetric interventions, and the detection of subtle forms of obstetric violence within health sciences curricula. Specifically, there is a need for content aimed at detecting subtle forms of obstetric violence in health science curricula, given the low level found. Future research should explore the cultural and contextual factors influencing the perception of obstetric violence.

## Figures and Tables

**Table 1:** Observed percentage of types of obstetric violence witnessed during academic training (*n* = 85).

As a student, I have observed…	Yes% (*n*)	No% (*n*)
Derogatory comments about women	70.6 (60)	29.4 (25)
Lack of time devoted to women’s care	61.2 (52)	38.8 (33)
Interventions without informed consent (e.g., medication administration, episiotomies, caesarean sections, etc.)	57.6 (49)	42.4 (36)
Lack of privacy for the woman	83.5 (71)	16.5 (14)
Women in labour being told they were endangering the life or health of their baby	10.6 (9)	89.4 (76)
Physical violence such as repeated vaginal examinations, aggressive physical contact, or procedures without anaesthesia	55.3 (47)	44.7 (38)
Women’s requests during childbirth being ignored	31.8 (27)	68.2 (58)
Use of the Kristeller manoeuvre to accelerate childbirth	45.9 (39)	54.1 (46)
Disclosure of private information without consent	9.4 (8)	90.6 (77)
Shouting at or infantilizing women in labour	32.9 (28)	67.1 (57)
Denial of desired treatments or encouragement to accept undesired interventions	27.1 (23)	72.9 (62)
What did you feel when witnessing these behaviours?	Yes% (*n*)	No% (*n*)
Cold sweats	11.8 (10)	88.2 (75)
Tachycardia	27.1 (23)	72.9 (62)
Difficulty concentrating	16.5 (14)	83.5 (71)
Anxiety	56.5 (48)	43.5 (37)
Trouble sleeping	10.6 (9)	89.4 (76)
Agitation	38.8 (33)	61.2 (52)
Hypervigilance	50.6 (43)	49.4 (42)
None of the above	4.7 (4)	95.3 (81)
Have you considered dropping out of school (temporarily) due to situations experienced during your internship?	20.0 (17)	80.0 (68)

Note: % = percentage; (*n*) = frequency.

**Table 2:** Relationship between the variables collected and the PercOV-S instrument.

	Visible OV ^1^			Invisible OV ^2^			Total OV ^3^		
	m (ds) ^4^	Mdn (IQR) ^5^	*p* ^6^	m (ds)^4^	Mdn (IQR) ^5^	*p* ^6^	m (ds) ^4^	Mdn (IQR) ^5^	*p* ^6^
Sociodemographic data									
Sex									
Female	2.92 (0.91)	3.00 (2.25–3.62)	0.005 *	4.30 (0.52)	4.44 (4.00–4.72)	0.000 *	3.97 (0.58)	4.09 (3.45–4.45)	0.000 *
Male	2.43 (0.81)	2.25 (1.87–2.87)		3.92 (0.53)	3.96 (3.54–4.34)		3.56 (0.55)	3.63 (3.16–3.92)	
Academic background									
Nursing	2.27 (0.93)	2.87 (1.87–3.50)	0.000 **	4.28 (0.51)	4.40 (3.96–4.68)	0.000 **	3.92 (0.58)	3.97 (3.51–4.24)	0.000 **
Medicine	2.76 (0.91)	2.62 (2.18–3.31)		4.04 (0.62)	4.28 (3.58–4.52)		3.73 (0.64)	3.78 (3.27–4.18)	
Psychology	2.73 (0.80)	2.75 (2.12–3.25)		4.15 (0.50)	4.32 (3.84–4.48)		3.81 (0.51)	3.93 (3.40–4.18)	
Midwifery Resident	3.73 (0.63)	3.87 (3.37–4.12)		4.68 (0.23)	4.76 (4.48–4.88)		4.45 (0.31)	4.54 (4.27–4.72)	
Other	2.51 (0.61)	2.37 (2.21–2.93)		4.05 (0.58)	4.12 (3.87–4.33)		3.67 (0.55)	3.67 (3.44–3.93)	
Obstetric data									
Have you ever had a pregnancy?									
Yes	3.29 (1.04)	3.37 (2.46–4.15)	0.011 *	4.45 (0.56)	4.62 (4.34–4.84)	0.010 *	4.17 (0.65)	4.30 (3.84–4.62)	0.006 *
No	2.82 (0.89)	2.87 (2.12–3.50)		4.24 (0.53)	4.34 (3.92–4.68)		3.90 (0.57)	3.97 (3.47–4.36)	
Have you ever given birth?									
Yes	3.34 (1.07)	3.50 (2.53–4.25)	0.017 *	4.44 (0.62)	4.66 (4.38–4.85)	0.026 *	4.17 (0.72)	4.34 (3.94–4.72)	0.014 *
No	2.83 (0.89)	2.87 (2.12–3.50)		4.25 (0.53)	4.36 (3.92–4.68)		3.91 (0.57)	3.97 (3.48–4.38)	
Do you think you have ever experienced a obstetric violence situation on a personal level?									
Yes	3.58 (0.72)	3.75 (3.09–4.15)	0.000 *	4.62 (0.31)	4.68 (4.48–4.84)	0.000 *	4.37 (0.39)	4.45 (4.14–4.69)	0.000 *
No	2.69 (0.87)	2.65 (2.00–3.75)		4.18 (0.54)	4.24 (3.84–4.64)		3.82 (0.58)	3.80 (3.39–4.27)	
Experience in childbirth									
Have you ever witnessed a woman giving birth?									
Yes	3.27 (0.93)	3.37 (2.62–4.00)	0.000 *	4.41 (0.55)	4.52 (4.28–4.80)	0.000 *	4.14 (0.61)	4.27 (3.84–4.57)	0.000 *
No	2.63 (0.82)	2.62 (2.00–3.25)		4.18 (0.51)	4.20 (3.84–4.62)		3.81 (0.54)	3.78 (3.40–4.24)	
Have you worked or completed clinical internships in the field of gynaecology and obstetrics?									
Yes	3.18 (0.96)	3.37 (2.40–4.00)	0.000 *	4.38 (0.54)	4.52 (4.17–4.78)	0.000 *	4.09 (0.61)	4.27 (3.73–4.57)	0.000 *
No	2.65 (0.82)	2.62 (2.03–3.25)		4.18 (0.51)	4.24 (3.88–4.64)		3.81 (0.54)	3.78 (3.42–4.24)	
Treatment during obstetric care									
Have all the women you’ve treated for obstetrically been treated the same?									
Yes	2.52 (0.86)	2.37 (1.72–3.12)	0.000 *	4.02 (0.60)	4.22 (3.45–4.49)	0.000 *	3.66 (0.62)	3.80 (2.96–4.10)	0.000 *
No	3.58 (0.77)	3.75 (3.05–4.12)		4.60 (0.36)	4.72 (4.44–4.84)		4.35 (0.44)	4.48 (4.09–4.69)	
Type of discrimination (n = 78)									
I think the discriminatory treatment I experienced was due to…									
Woman’s weight									
Yes	3.86 (0.65)	4.00 (3.37–4.25)	0.009 *	4.76 (0.17)	4.80 (4.66–4.88)	0.009 *	4.54 (0.27)	4.60 (4.35–4.75)	0.005 *
No	3.43 (0.81)	3.50 (2.75–4.00)		4.51 (0.41)	4.56 (4.28–4.84)		4.25 (0.49)	4.30 (3.77–4.63)	
Woman’s opinion									
Yes	3.73 (0.75)	4.00 (3.37–4.18)	0.114 *	4.72 (0.26)	4.84 (4.54–4.88)	0.032 *	4.48 (0.36)	4.60 (4.30–4.74)	0.038 *
No	3.52 (0.79)	3.62 (2.87–4.00)		4.54 (0.40)	4.64 (4.36–4.82)		4.29 (0.47)	4.36 (4.00–4.62)	
Her age									
Yes	3.78 (0.75)	4.00 (3.32–4.25)	0.104 *	4.71 (0.27)	4.80 (4.64–4.93)	0.080 *	4.48 (0.37)	4.59 (4.23–4.75)	0.049 *
No	3.51 (0.79)	3.62 (2.95–4.00)		4.56 (0.39)	4.66 (4.38–4.84)		4.30 (0.46)	4.37 (4.05–4.65)	
Health and gender									
Have you ever heard of the obstetric violence concept?									
Yes	3.02 (0.94)	3.12 (2.12–3.87)	0.000 *	4.36 (0.52)	4.48 (3.96–4.76)	0.000 *	4.03 (0.58)	4.15 (3.56–4.51)	0.000 *
No	2.33 (0.61)	2.37 (1.75–2.75)		3.93 (0.48)	3.96 (3.51–4.28)		3.54 (0.45)	3.48 (3.12–3.87)	
Treatment of obstetric violence									
Do you agree with the term “Obstetric Violence”?									
Yes	2.93 (0.91)	3.00 (2.12–3.62)	0.003 *	4.32 (0.48)	4.40 (3.88–4.72)	0.000 *	3.99 (0.55)	4.09 (3.45–4.45)	0.000 *
No	2.44 (0.89)	2.25 (1.72–2.93)		3.84 (0.73)	4.00 (3.12–4.38)		3.50 (0.74)	3.48 (2.87–3.98)	
Do you think the term “Obstetric Violence” criminalizes healthcare professionals?									
Yes	2.52 (0.89)	2.43 (1.67–3.12)	0.000 *	4.01 (0.59)	4.04 (3.53–4.41)	0.000 *	3.65 (0.61)	3.60 (3.14–4.05)	0.000 *
No	3.02 (0.90)	3.00 (2.12–3.75)		4.37 (0.48)	4.48 (3.96–4.72)		4.04 (0.55)	4.12 (3.54–4.48)	
Obstetric violence during training									
Do you think you have witnessed any obstetric violence situations?									
Yes	3.57 (0.71)	3.75 (2.87–4.12)	0.000 *	4.61 (0.33)	4.68 (4.43–4.84)	0.000 *	4.36 (0.40)	4.48 (4.00–4.69)	0.000 *
No	2.60 (0.85)	2.50 (1.82–3.25)		4.13 (0.55)	4.20 (3.68–4.56)		3.76 (0.57)	3.75 (3.24–4.21)	
Type of obstetric violence witnessed during training “As a student I have observed…” (n = 85)									
Non-informed consent interventions (medication administration, episiotomies, caesarean sections, etc.)									
Yes	3.78 (0.67)	4.00 (3.37–4.25)	0.001 *	4.67 (0.32)	4.76 (4.48–4.88)	0.020 *	4.46 (0.39)	4.57 (4.24–4.75)	0.003 *
No	3.29 (0.36)	3.25 (2.62–3.87)		4.53 (0.32)	4.62 (4.36–4.76)		4.23 (0.38)	4.30 (3.93–4.54)	
Women who were giving birth were told that they were putting their baby’s life or health at risk.									
Yes	3.96 (0.69)	4.12 (3.52–4.50)	0.050 *	4.78 (0.24)	4.84 (4.68–4.92)	0.053 *	4.58 (0.33)	4.72 (4.41–4.75)	0.030 *
No	3.52 (0.70)	3.75 (2.87–4.03)		4.59 (0.33)	4.66 (4.40–4.84)		4.33 (0.40)	4.39 (4.00–4.61)	
Physical violence such as: repeated vaginal examinations, aggressive physical contact, interventions without anaesthesia, etc.									
Yes	3.76 (0.62)	3.87 (3.15–4.12)	0.015 *	4.74 (0.20)	4.80 (4.48–4.90)	0.000 *	4.50 (0.28)	4.57 (4.23–4.74)	0.001 *
No	3.34 (0.75)	3.37 (2.50–3.96)		4.46 (0.39)	4.52 (4.16–4.72)		4.19 (0.45)	4.27 (3.83–4.54)	
Kristeller manoeuvre to accelerate labour									
Yes	3.73 (0.61)	3.87 (3.32–4.12)	0.055 *	4.67 (0.34)	4.76 (4.48–4.88)	0.075 *	4.44 (0.39)	4.54 (4.21–4.71)	0.041 *
No	3.43 (0.76)	3.37 (2.62–4.00)		4.57 (0.31)	4.64 (4.28–4.84)		4.29 (0.39)	4.33 (3.93–4.60)	
Women were denied treatment they wanted or encouraged to accept interventions they did not want									
Yes	3.81 (0.68)	4.00 (3.30–4.37)	0.033 *	4.69 (0.24)	4.72 (4.45–4.90)	0.308 *	4.48 (0.33)	4.57 (4.19–4.75)	0.807 *
No	3.48 (0.70)	3.56 (2.87–4.00)		4.59 (0.35)	4.68 (4.40–4.84)		4.32 (0.41)	4.39 (4.00–4.60)	
What did you feel when you witnessed these dealings?									
Trouble sleeping									
Yes	4.21 (0.24)	4.25 (4.00–4.37)	0.002 *	4.82 (0.15)	4.80 (4.66–4.92)	0.031 *	4.67 (0.13)	4.69 (4.53–4.75)	0.007 *
No	3.50 (0.71)	3.50 (2.87–4.03)		4.59 (0.34)	4.66 (4.40–4.84)		4.32 (0.40)	4.36 (4.00–4.61)	
Agitation									
Yes	3.80 (0.60)	4.00 (3.30–4.25)	0.014 *	4.70 (0.26)	4.80 (4.48–4.88)	0.048 *	4.48 (0.33)	4.57 (4.23–4.75)	0.014 *
No	3.43 (0.74)	3.37 (2.62–4.00)		4.56 (0.35)	4.64 (4.36–4.81)		4.28 (0.42)	4.31 (3.94–4.60)	
None of the above									
Yes	3.09 (0.41)	3.12 (2.77–3.40)	0.148 *	4.34 (0.24)	4.42 (4.21–4.49)	0.040 *	4.04 (0.26)	4.10 (3.92–4.15)	0.045 *
No	3.59 (0.71)	3.75 (2.87–4.12)		4.63 (0.33)	4.72 (4.44–4.84)		4.38 (0.39)	4.48 (4.00–4.69)	

Note: ^1^ protocolized–visible obstetric violence dimension; ^2^ non-protocolized–invisible obstetric violence dimension; ^3^ PercOV-S total; ^4^ mean (standard deviation); ^5^ median (interquartile range); ^6^ *p*-value; * Mann–Whitney U; ** Kruskal–Wallis.

**Table 3:** Relationship between sex and academic background with collected variables.

	Sex			Academic Background					
	Female	Male		Nursing	Medicine	Psychology	Midwifery Resident	Other	
	% (*n*)	% (*n*)	*p*	% (*n*)	% (*n*)	% (*n*)	% (*n*)	% (*n*)	*p*
Treatment of obstetric violence									
Do you agree with the term “Obstetric Violence”?									
Yes	90.8 (248)	67.7 (21)	0.001 **	92.5 (149)	70.2 (33)	90.7 (39)	91.9 (34)	87.5 (14)	0.002 **
No	9.2 (25)	32.3 (10)		7.5 (12)	29.8 (14)	9.3 (4)	8.1 (3)	12.5 (2)	
Do you think the term “Obstetric Violence” criminalizes healthcare professionals?									
Yes	27.1 (74)	45.2 (14)	0.035 *	23.0 (37)	53.2 (25)	25.6 (11)	16.2 (6)	56.3 (9)	0.000 *
No	72.9 (199)	54.8 (17)		77.0 (124)	46.8 (22)	74.4 (32)	83.8 (31)	43.8 (7)	
Treatment during obstetric care									
Have all the women you’ve treated for obstetrically been treated the same? (n = 126)									
Yes	33.3 (38)	83.3 (10)	0.001 *	47.7 (31)	60.9 (14)	100 (1)	5.4 (2)	-	0.001 **
No	66.7 (76)	16.7 (2)		52.3 (34)	39.1 (9)	-	94.6 (35)	-	
Obstetric violence during training									
Do you think you have witnessed any obstetric violence situations?									
Yes	30.0 (82)	9.7 (3)	0.016 *	24.8 (40)	14.9 (7)	7.0 (3)	94.6 (35)	-	0.000 *
No	70.0 (191)	90.3 (28)		75.2 (121)	85.1 (40)	93.0 (40)	5.4 (2)	100 (16)	
Type of obstetric violence witnessed during training “As a student I have observed…” (n = 85)									
Non- informed consent interventions (medication administration, episiotomies, caesarean sections, etc.)									
Yes	59.8 (49)	-	0.072 **	37.5 (15)	57.1 (4)	33.3 (1)	82.9 (29)	-	0.000 **
No	40.2 (33)	100 (3)		62.5 (25)	42.9 (3)	66.7 (2)	17.1 (6)	-	
Physical violence such as: repeated vaginal examinations, aggressive physical contact, interventions without anaesthesia, etc.									
Yes	56.1 (46)	33.3 (1)	0.584 **	40.0 (16)	57.1 (4)	33.3 (1)	74.3 (26)	-	0.013 **
No	43.9 (36)	66.7 (2)		60.0 (24)	42.9 (3)	66.7 (2)	25.7 (9)	-	
Kristeller manoeuvre to accelerate labour									
Yes	47.6 (39)	-	0.246 **	22.5 (9)	42.9 (3)	33.3 (1)	74.3 (26)	-	0.000 **
No	51.4 (43)	100 (3)		77.5 (31)	57.1 (4)	66.7 (2)	25.7 (9)	-	
Women were denied treatment they wanted or encouraged to accept interventions they did not want									
Yes	28.0 (23)	-	0.559 **	7.5 (3)	14.3 (1)	-	54.3 (19)	-	0.000 **
No	72.0 (59)	100 (3)		92.5 (37)	85.7 (6)	100 (3)	45.7 (16)	-	

Note: % (*n*): frequencies; *p*: *p*-value; * Chi square; ** Fisher.

## Data Availability

Dataset available on request from the authors. The data are not publicly available due to privacy and ethical restrictions.

## Supplementary Materials

The following supporting information can be downloaded at https://www.mdpi.com/article/10.3390/nursrep15120425/s1, Supplementary Material S1: Descriptive results and differences observed between the overall score of the PercOV-S instrument and its dimensions and the variables included in the study were not significant; Supplementary Material S2: Descriptive results and observed differences of the sociodemographic variables with the rest of the variables included in the study.

## Public Involvement Statement

No public involvement in any aspect of this research.

## Guidelines and Standards Statement

This manuscript was drafted against the STROBE for observational studies.
