# Supplementary material for: Factors Associated with the Perception of Obstetric Violence and Its Emotional Impact on Healthcare Training: A Cross-Sectional Study
Source: Nurs Rep. 2025 Nov 28;15(12):425. doi: 10.3390/nursrep15120425 (PMC12735495; doi:10.3390/nursrep15120425)
Supplement: Supplementary file 1 [file nursrep-15-00425-s001.zip › nursrep-3975938-Supplementary Material S1.pdf]

Supplementary material S1. Descriptive results and differences observed between the overall score of the PercOV-S instrument and its dimensions and the variables included in the study were not significant.

	Visible OV[1]			Invisible OV[2]			Total OV[3]		
	m(ds)[4]	Mdn(IQR)[5]	p[6]	m(ds)[4]	Mdn(IQR)[5]	p[6]	m(ds)[4]	Mdn(IQR)[5]	p[6]
Sociodemographic data									
Academic year									
1	2.87(0.918)	2.87(1.97-3.68)	0.162**	4.23(0.544)	4.32(3.76-4.68)	0.432**	3.90(0.595)	4.00(3.38-4.33)	0.256**
2	2.74(0.861)	2.62(1.87-3.37)		4.22(0.502)	4.28(3.80-4.60)		3.86(0.547)	3.84(3.39-4.30)	
3	2.76(0.961)	2.75(1.87-3.50)		4.23(0.666)	4.40(3.80-4.72)		3.87(0.697)	3.78(3.38-4.45)	
4	2.97(0.908)	3.12(2.10-3.50)		4.34(0.478)	4.40(3.92-4.72)		4.01(0.549)	4.09(3.53-4.42)	
5	3.07(0.758)	3.00(2.55-3.25)		4.42(0.261)	4.48(4.35-4.48)		4.09(0.345)	4.09(3.88-4.18)	
6	3.36(1.064)	3.36(2.30-4.34)		4.42(0.514)	4.60(4.28-4.75)		4.17(0.618)	4.27(3.85-4.66)	
You were born in Spain									
Yes	2.89(0.918)	2.87(2.00-3.62)	0.184*	4.27(0.540)	4.40(3.84-4.68)	0.503*	3.93(0.593)	4.00(3.42-4.24)	0.338*
No	2.58(0.940)	2.50(1.87-3.25)		4.19(0.534)	4.40(3.69-4.60)		3.80(0.589)	3.81(3.25-4.27)	
Treatment during obstetric care									
When you have entered a room or ward for obstetric care, have you or the person supervising the woman asked her permission?									
Yes	3.06(1.00)	3.25(2.00-3.87)	0.163**	4.31(0.61)	4.48(3.80-4.76)	0.369**	4.00(0.68)	4.16(3.39-4.54)	0.232**
No	3.24(1.07)	3.37(2.27-3.87)		4.52(0.27)	4.52(4.34-4.68)		4.21(0.43)	4.30(3.87-4.30)	
Sometimes	3.53(0.71)	3.62(2.95-4.00)		4.56(0.34)	4.64(4.40-4.80)		4.31(0.41)	4.39(4.07-4.60)	
Almost never	3.48(0.87)	3.56(2.62-4.12)		4.57(0.39)	4.64(4.28-4.87)		4.30(0.49)	4.33(3.97-4.74)	
Type of discrimination (n=78)									
Her origin. skin colour, language, or cultural background									
Yes	3.60(0.86)	3.87(3.02-4.18)	0.613*	4.61(0.37)	4.76(4.44-4.84)	0.924*	4.36(0.46)	4.51(4.09-4.72)	0.660*
No	3.58(0.70)	3.75(3.07-4.00)		4.60(0.36)	4.64(4.42-4.88)		4.35(0.43)	4.48(4.10-4.69)	
Her social status									
Yes	3.70(0.65)	3.87(3.12-4.12)	0.474*	4.65(0.28)	4.72(4.40-4.84)	0.927*	4.42(0.34)	4.53(4.09-4.73)	0.633*
No	3.54(0.83)	3.75(2.82-4.12)		4.59(0.40)	4.72(4.38-4.87)		4.33(0.48)	4.48(4.07-4.69)	
A disability or pre-existing condition									
Yes	4.08(0.80)	3.75(3.62-4.37)	0.471*	4.79(0.24)	4.84(4.68-4.92)	0.314*	4.62(0.37)	4.57(4.42-4.78)	0.409*
No	3.57(0.78)	3.75(3.25-4.12)		4.60(0.37)	4.72(4.44-4.84)		4.35(0.44)	4.48(4.16-4.69)	
Her sexual orientation									
Yes	3.79(0.36)	4.00(3.62-4.00)	0.769*	4.68(0.34)	4.72(4.48-4.86)	0.826*	4.46(0.34)	4.54(4.27-4.65)	0.788*
No	3.58(0.79)	3.75(2.97-4.12)		4.60(0.37)	4.72(4.44-4.84)		4.35(0.45)	4.48(4.09-4.69)	
No apparent reason									
Yes	3.72(0.68)	3.87(3.35-4.12)	0.270*	4.63(0.37)	4.76(4.43-4.88)	0.435*	4.41(0.43)	4.54(4.24-4.69)	0.395*
No	3.49(0.85)	3.50(2.87-4.12)		4.58(0.36)	4.68(4.44-4.84)		4.32(0.46)	4.36(3.94-4.71)	
Health and gender									
During your academic training, have you had any subjects or topics where the gender perspective was incorporated?									
Yes	2.83(0.91)	2.75(2.12-3.5)	0.127*	4.27(0.53)	4.36(3.96-4.68)	0.944*	3.92(0.58)	3.95(3.50-4.39)	0.614*
No	3.02(0.94)	3.00(2.34-3.75)		4.25(0.57)	4.40(3.88-4.68)		3.95(0.62)	4.09(3.50-4.48)	
Type of obstetric violence witnessed during training "As a student I have observed…"									
Derogatory comments about women									
Yes	3.58(0.70)	3.75(3.09-4.12)	0.923*	4.62(0.35)	4.72(4.44-4.85)	0.556*	4.37(0.41)	4.48(4.11-4.69)	0.710*
No	3.56(0.73)	3.75(3.12-4.12)		4.60(0.29)	4.68(4.44-4.80)		4.35(0.37)	4.36(4.15-4.63)	
Lack of time devoted to women's care									
Yes	3.54(0.71)	3.75(2.96-4.12)	0.708*	4.63(0.29)	4.70(4.44-4.84)	0.932*	4.37(0.36)	4.48(4.06-4.67)	0.889*
No	3.61(0.71)	3.75(3.25-4.12)		4.59(0.39)	4.68(4.48-4.84)		4.35(0.45)	4.48(4.18-4.69)	
Lack of privacy for the woman									
Yes	3.63(0.68)	3.87(3.25-4.12)	0.141*	4.66(0.25)	4.72(4.48-4.84)	0.094*	4.41(0.33)	4.48(4.24-4.69)	0.100*
No	3.29(0.80)	3.31(2.62-3.84)		4.39(0.54)	4.44(4.16-4.81)		4.12(0.59)	4.15(3.81-4.59)	
Women's requests during childbirth being ignored									
Yes	3.67(0.74)	3.87(3.18-4.31)	0.276*	4.71(0.21)	4.76(4.62-4.86)	0.138*	4.46(0.32)	4.51(4.30-4.72)	0.163*
No	3.52(0.70)	3.56(3.12-4.09)		4.57(0.36)	4.64(4.44-4.84)		4.32(0.42)	4.36(4.09-4.62)	

Disclosure of private information without consent									
Yes	3.53(0.78)	3.93(3.06-4.03)	0.940*	4.63(0.45)	4.84(4.39-4.96)	0.407*	4.36(0.52)	4.62(4.06-4.75)	0.562*
No	3.57(0.71)	3.75(3.12-4.12)		4.61(0.32)	4.68(4.44-4.84)		4.36(0.38)	4.48(4.12-4.66)	
Shouting at or infantilizing women in labour									
Yes	3.79(0.65)	4.00(3.46-4.25)	0.058*	4.66(0.26)	4.72(4.47-4.85)	0.530*	4.45(0.33)	4.50(4.30-4.70)	0.225*
No	3.46(0.71)	3.37(2.87-4.12)		4.59(0.36)	4.68(4.44-4.84)		4.32(0.42)	4.36(4.00-4.66)	
What did you feel when witnessing these behaviours?									
Cold sweats									
Yes	3.55(0.85)	3.62(2.96-4.21)	0.886*	4.74(0.22)	4.76(4.61-4.95)	0.199*	4.45(0.35)	4.45(4.29-4.75)	0.413*
No	3.57(0.69)	3.75(3.12-4.12)		4.60(0.34)	4.68(4.44-4.84)		4.35(0.40)	4.48(4.10-4.66)	
Tachycardia									
Yes	3.63(0.64)	3.87(3.06-4.06)	0.688*	4.64(0.28)	4.76(4.46-4.86)	0.763*	4.40(0.34)	4.48(4.13-4.68)	0.755*
No	3.55(0.74)	3.68(3.15-4.12)		4.60(0.35)	4.66(4.44-4.84)		4.35(0.42)	4.42(4.12-4.68)	
Difficulty concentrating									
Yes	3.70(0.61)	4.00(3.37-4.00)	0.426**	4.67(0.25)	4.74(4.52-4.83)	0.704*	4.43(0.30)	4.50(4.31-4.65)	0.627*
No	3.55(0.73)	4.42(4.10-4.69)		4.60(0.34)	4.68(4.44-4.84)		4.35(0.41)	4.42(4.10-4.69)	
Anxiety									
Yes	3.57(0.70)	3.75(3.18-4.12)	0.940*	4.63(0.30)	4.72(4.44-4.85)	0.588*	4.38(0.37)	4.48(4.17-4.67)	0.716*
No	3.57(0.73)	3.75(3.12-4.12)		4.59(0.36)	4.68(4.44-4.84)		4.34(0.43)	4.51(4.09-4.67)	
Hypervigilance									
Yes	3.70(0.71)	3.87(3.25-4.12)	0.096*	4.63(0.35)	4.72(4.54-4.84)	0.470*	4.40(0.41)	4.54(4.27-4.69)	0.181*
No	3.44(0.68)	3.37(2.87-4.00)		4.60(0.31)	4.60(4.44-4.87)		4.32(0.38)	4.30(4.09-4.62)	
Have you considered dropping out of school (temporarily) due to situations experienced during your internship?									
Yes	3.69(0.79)	4.00(3.12-4.37)	0.303*	4.69(0.22)	4.72(4.64-4.84)	0.458**	4.45(0.33)	4.54(4.27-4.75)	0.289*
No	3.54(0.69)	3.75(3.09-4.03)		4.59(0.35)	4.66(4.44-4.84)		4.34(0.41)	4.39(4.11-4.64)	

Note: [1] *protocolized–visible obstetric violence dimension;* [2] *non-protocolized–invisible obstetric violence dimension;* [3] *PercOV-S total;* [4] *mean (standard deviation);* [5] *median (interquartile range);* [6]*p-value; * Mann–Whitney U; **Kruskal–Wallis.*
