# Supplementary material for: Factors Associated with the Perception of Obstetric Violence and Its Emotional Impact on Healthcare Training: A Cross-Sectional Study
Source: Nurs Rep. 2025 Nov 28;15(12):425. doi: 10.3390/nursrep15120425 (PMC12735495; doi:10.3390/nursrep15120425)
Supplement: Supplementary file 1 [file nursrep-15-00425-s001.zip › nursrep-3975938-Supplementary Material S2.pdf]

Supplementary material S2. Descriptive results and observed differences of the sociodemographic variables with the rest of the variables included in the study

	Sex			Academic background					
	**Male**	**Female**		**Nursing**	**Medicine**	**Psychology**	**Midwife resident**	**Others**	
	**%(n)**	**%(n)**	**p**	**%(n)**	**%(n)**	**%(n)**	**%(n)**	**%(n)**	**p**
Experience in childbirth									
**Have you ever witnessed a woman giving birth?**									
Yes	67.7(21)	62.3(170)	0.550*	33.5(54)	44.7(21)	2.3(1)	97.3(36)	6.3(1)	0.000*
No	32.3(10)	37.7(103)		66.5(107)	55.3(26)	97.7(42)	2.7(1)	93.8(15)	
**Have you worked or completed clinical internships in the field of gynaecology and obstetrics?**									
Yes	38.7(12)	61.3(19)	0.744*	40.4(65)	48.9(23)	2.3(1)	100(37)	-	0.000*
No	41.8(114)	58.2(159)		59.6(96)	51.1(24)	97.7(42)	-	100(16)	
Treatment during obstetric care									
**When you have entered a room or ward for obstetric care, have you or the person supervising the woman asked her permission?**									
Yes	75.0(9)	67.5(77)	0.726**	60.0(39)	82.6(19)	100(1)	73.0(27)	-	0.268**
No	-	7.9(9)		12.3(8)	-	-	2.7(1)	-	
Sometimes	16.7(2)	20.2(23)		23.1(15)	8.7(2)	-	21.6(8)	-	
Almost never	8.3(1)	4.4(5)		4.6(3)	8.7(2)	-	2.7(1)	-	
**Have all the women you've treated for obstetrically been treated the same?**									
Yes	83.3(10)	33.3(38)	0.001**	47.7(31)	60.9(14)	100(1)	5.4(2)	-	0.001**
No	16.7(2)	66.7(76)		52.3(34)	39.1(9)	-	94.6(35)	-	
Type of discrimination (n=78)									
**Woman's weight**									
Yes	-	38.2(29)	0.526**	20.6(7)	33.3(3)	-	54.3(19)	-	0.012**
No	100(2)	61.8(47)		79.4(27)	66.7(6)	-	45.7(16)	-	
**Woman's opinion**									
Yes	-	35.5(27)	0.541**	26.5(9)	33.3(3)	-	42.9(15)	-	0.394**
No	100(2)	64.5(49)		73.5(25)	66.7(6)	-	57.1(20)	-	
**Her origin. skin colour, language, or cultural background**									
Yes	50.0(1)	50.0(38)	1.000**	44.1(15)	44.4(4)	-	57.1(20)	-	0.536**
No	50.0(1)	50.0(38)		55.9(19)	55.6(5)	-	42.9(15)	-	
**Her age**									
Yes	50.0(1)	30.3(23)	0.523**	32.4(11)	22.2(2)	-	31.4(11)	-	0.886**
No	50.0(1)	69.7(53)		67.6(23)	77.8(7)	-	68.6(24)	-	
**Her social status**									
Yes	50.0(1)	30.3(23)	0.523**	26.5(9)	33.3(3)	-	34.3(12)	-	0.781**
No	50.0(1)	69.7(53)		73.5(25)	66.7(6)	-	65.7(23)	-	
**A disability or pre-existing condition**									
Yes	-	3.9(3)	1.000**	-	22.2(2)	-	2.9(1)	-	0.033**
No	100(2)	96.1(73)		100(34)	77.8(7)	-	97.1(34)	-	
**Her sexual orientation**									
Yes	-	3.9(3)	1.000**	5.9(2)	11.1(1)	-	-	-	0.100**
No	100(2)	96.1(73)		94.1(32)	88.9(8)	-	100(35)	-	
**No apparent reason**									
Yes	50.0(1)	44.7(34)	1.000**	-	48.6(17)	-	48.6(17)	-	0.897**
No	50.0(1)	55.3(42)		100(35)	51.4(18)	-	51.4(18)	-	
Health and gender									
**Have you ever heard of the obstetric violence concept?**									
Yes	71.0(22)	79.5(217)	0.273*	81.4(131)	80.9(38)	58.1(25)	100(37)	50.0(8)	0.000*
No	29.0(9)	20.5(56)		18.6(30)	19.1(9)	41.9(18)	-	50.0(8)	
Type of obstetric violence witnessed during training "As a student I have observed…" (n=85)									
**Derogatory comments about women**									

Yes	33.3(1)	66.7(2)	0.205**	62.5(25)	42.9(3)	-	82.9(29)	-	0.051**
No	72.0(59)	28.0(23)		37.5(15)	57.1(4)	100(3)	17.1(6)	-	
**Lack of time devoted to women's care**									
Yes	33.3(1)	66.7(2)	0.556**	62.5(25)	57.1(4)	66.7(2)	60.0(21)	-	1.000**
No	62.2(51)	37.8(31)		37.5(15)	42.9(3)	33.3(1)	40.0(14)	-	
**Lack of privacy for the woman**									
Yes	100(3)	82.9(68)	1.000**	75.0(30)	100(7)	66.7(2)	91.4(32)	-	0.113**
No	-	17.1(14)		25.0(10)	-	33.3(1)	8.6(3)	-	
**Women in labour being told they were endangering the life or health of their baby**									
Yes	-	11.0(9)	1.000**	2.5(1)	-	-	22.9(8)	-	0.034**
No	100(3)	89.0(73)		97.5(39)	100(7)	100(3)	77.1(27)	-	
**Women's requests during childbirth being ignored**									
Yes	33.3(1)	31.7(26)	1.000**	27.5(11)	28.6(2)	66.7(2)	34.3(12)	-	0.555**
No	66.7(2)	68.3(56)		72.5(29)	71.4(5)	33.3(1)	65.7(23)	-	
**Disclosure of private information without consent**									
Yes	-	100(3)	1.000*	7.5(3)	28.6(2)	-	8.6(3)	-	0.371**
No	100(3)	90.2(74)		92.5(37)	71.4(5)	100(3)	91.4(32)	-	
**Shouting at or infantilizing women in labour**									
Yes	-	100(3)	0.547**	25.0(10)	14.3(1)	-	48.6(17)	-	0.069**
No	34.1(28)	65.9(54)		75.0(30)	85.7(6)	100(3)	51.4(18)	-	
**What did you feel when witnessing these behaviours?**									
**Cold sweats**									
Yes	-	12.2(10)	1.000**	10.0(4)	14.3(1)	-	14.3(5)	-	0.854**
No	100(3)	87.8(72)		90.0(36)	85.7(6)	100(3)	85.7(30)	-	
**Tachycardia**									
Yes	-	28.0(23)	0.559**	20.0(8)	57.1(4)	-	31.4(11)	-	0.132**
No	100(3)	72.0(59)		80.0(32)	42.9(3)	100(3)	68.6(24)	-	
**Difficulty concentrating**									
Yes	33.3(1)	15.9(13)	0.421**	7.5(3)	42.9(3)	-	22.9(8)	-	0.050**
No	66.7(2)	84.1(69)		92.5(37)	57.1(4)	100(3)	77.1(27)	-	
**Anxiety**									
Yes	-	58.5(48)	0.078**	50.0(20)	57.1(4)	-	60.0(21)	-	0.461**
No	100(3)	41.5(34)		50.0(20)	42.9(3)	100(3)	40.0(14)	-	
**Trouble sleeping**									
Yes	-	11.0(9)	1.000**	10.0(4)	-	-	14.3(5)	-	0.761**
No	100(3)	89.0(73)		90.0(36)	100(7)	100(3)	85.7(30)	-	
**Agitation**									
Yes	-	40.2(33)	0.278**	22.9(9)	42.9(3)	-	57.1(20)	-	0.011**
No	100(3)	59.8(49)		77.5(31)	57.1(4)	100(3)	42.9(15)	-	
**Hypervigilance**									
Yes	-	52.4(43)	0.116**	55.0(22)	57.1(4)	-	48.6(17)	-	0.377**
No	100(3)	47.6(39)		45.0(18)	42.9(3)	100(3)	51.4(18)	-	
**None of the above**									
Yes	66.7(2)	2.4(2)	0.005**	5.0(2)	14.3(1)	-	2.9(1)	-	0.470**
No	33.3(1)	97.6(80)		95.0(38)	85.7(6)	100(3)	97.1(34)	-	
**Have you considered dropping out of school (temporarily) due to situations experienced during your internship?**									
Yes	-	**20.7(17)**	1.000**	17.5(7)	57.1(4)	-	17.1(6)	-	0.114**
No	100(3)	**79.3(65)**		82.5(33)	42.9(3)	100(3)	82.9(29)	-	

Note: % = Percentage; (n) = Frequency; p = p-value; *Chi Square; **Fisher

Supplementary material S2. Descriptive results and observed differences of the sociodemographic variables with the rest of the variables included in the study

	**You were born in Spain**			**Academic year**						
	**Yes**	**No**		**1**	**2**	**3**	**4**	**5**	**6**	
	**%(n)**	**%(n)**	**p**	**%(n)**	**%(n)**	**%(n)**	**%(n)**	**%(n)**	**%(n)**	**p**
Experience in childbirth										
**Have you ever witnessed a woman giving birth?**										
Yes	35.9(103)	58.8(10)	0.057*	28.0(21)	27.9(24)	25.5(14)	55.4(36)	60.0(3)	83.3(15)	0.000*
No	64.1(184)	41.2(7)		72.0(54)	72.1(62)	74.5(41)	44.6(29)	40.0(2)	16.7(3)	
**Have you worked or completed clinical internships in the field of gynaecology and obstetrics?**										
Yes	40.4(116)	58.8(10)	0.134*	26.7(20)	30.2(26)	34.5(19)	63.1(41)	80.0(4)	88.9(16)	0.000*
No	59.6(171)	41.2(7)		73.3(55)	69.8(60)	65.5(36)	36.9(24)	20.0(1)	11.1(2)	
Treatment during obstetric care (n = 126)										
**When you have entered a room or ward for obstetric care, have you or the person supervising the woman asked her permission?**										
Yes	69.8(81)	50.0(5)	0.058**	85.0(17)	76.9(20)	63.1(12)	56.1(23)	50.0(2)	75.0(12)	0.152*
No	5.2(6)	30.0(3)		5.0(1)	7.7(2)	-	12.2(5)	25.0(1)	-	
Sometimes	19.8(23)	20.0(2)		10.0(2)	7.7(2)	31.6(6)	29.3(12)	-	18.8(3)	
Almost never	5.2(6)	-		-	7.7(2)	5.3(1)	2.4(1)	25.0(1)	6.3(1)	
**Have all the women you've treated for obstetrically been treated the same?**										
Yes	37.1(43)	50.0(5)	0.503**	20.0(4)	46.2(12)	36.8(7)	41.5(17)	75.0(3)	31.3(5)	0.275*
No	62.9(73)	50.0(5)		80.0(16)	53.8(14)	63.2(12)	58.5(24)	25.0(1)	68.8(11)	
Type of discrimination (n=78)										
**I think the discriminatory treatment I experienced was due to…**										
**Woman's weight**										
Yes	37.0(27)	40.0(2)	1.000**	37.5(6)	57.1(8)	8.3(1)	29.2(7)	100(1)	54.5(6)	0.048**
No	63.0(46)	60.0(3)		62.5(10)	42.9(6)	91.7(11)	70.8(17)	-	45.5(5)	
**Woman's opinion**										
Yes	35.6(26)	20.0(1)	0.654**	31.3(5)	42.9(6)	25.0(3)	29.2(7)	-	54.5(6)	0.621**
No	64.4(47)	80.0(4)		68.8(11)	57.1(8)	75.0(9)	70.8(17)	100(1)	45.5(5)	
**Her origin. skin colour, language, or cultural background**										
Yes	50.7(37)	40.0(2)	1.000**	50.0(8)	57.1(8)	16.7(2)	62.5(15)	-	54.5(6)	0.145*
No	49.3(36)	60.0(3)		50.0(8)	42.9(6)	83.3(10)	37.5(9)	100(1)	45.5(5)	
**Her age**										
Yes	32.9(24)	-	0.316**	31.3(5)	42.9(6)	25.0(3)	33.3(8)	-	18.2(2)	0.831**
No	67.1(49)	100(5)		68.8(11)	57.1(8)	75.0(9)	66.7(16)	100(1)	81.8(9)	
**Her social status**										
Yes	32.9(24)	-	0.316**	12.5(2)	50.0(7)	25.0(3)	33.3(8)	-	36.4(4)	0.314**
No	67.1(49)	100(5)		87.5(14)	50.0(7)	75.0(9)	66.7(16)	100(1)	63.6(7)	
**A disability or pre-existing condition**										
Yes	4.1(3)	-	1.000**	-	7.1(1)	-	4.2(1)	-	9.1(1)	0.651**
No	95.9(70)	100(5)		100(6)	92.9(13)	100(12)	95.8(23)	100(1)	90.9(10)	
**Her sexual orientation**										
Yes	4.1(3)	-	1.000**	-	-	8.3(1)	4.2(1)	-	9.1(1)	0.559**
No	95.9(70)	100(5)		100(16)	100(14)	91.7(11)	95.8(23)	100(1)	90.9(10)	
**No apparent reason**										
Yes	43.8(32)	60.0(3)	0.652**	62.5(10)	28.6(4)	58.3(7)	37.5(9)	100(1)	36.4(4)	0.258**
No	56.2(41)	40.0(2)		37.4(6)	71.4(10)	41.7(5)	62.5(15)	-	63.6(7)	
Health and gender										
**Have you ever heard of the obstetric violence concept?**										
Yes	79.4(228)	64.7(11)	0.217**	66.7(50)	79.1(68)	74.5(41)	87.7(57)	100(5)	100(18)	0.004**

No	20.6(59)	35.3(6)		33.3(25)	20.9(18)	25.5(14)	12.3(8)	-	-	
Treatment of obstetric violence										
**Do you agree with the term "Obstetric Violence"?**										
Yes	87.8(252)	90.8(248)	0.235**	85.3(64)	89.5(77)	90.9(50)	93.8(61)	60.0(3)	77.8(14)	0.103**
No	12.2(35)	9.2(25)		14.7(11)	10.5(9)	9.1(5)	6.2(4)	40.0(2)	22.2(4)	
**Do you think the term "Obstetric Violence" criminalizes healthcare professionals?**										
Yes	29.6(85)	17.6(3)	0.411**	26.7(20)	33.7(29)	32.7(18)	23.1(15)	40.0(2)	22.2(4)	0.652**
No	70.4(202)	82.4(14)		73.3(55)	66.3(57)	67.3(37)	76.9(50)	60.0(3)	77.8(14)	
Obstetric violence during training										
**Do you think you have witnessed any obstetric violence situations?**										
Yes	27.9(80)	29.4(5)	1.000**	26.7(20)	23.3(20)	21.8(12)	33.8(22)	60.0(3)	44.4(8)	0.149*
No	72.1(207)	70.6(12)		73.3(55)	76.7(66)	78.2(43)	66.2(43)	40.0(2)	55.6(10)	
Type of obstetric violence witnessed during training "As a student I have observed…" (n=85)										
**Derogatory comments about women**										
Yes	73.8(59)	20.0(1)	0.024**	80.0(16)	75.0(15)	58.3(7)	68.2(15)	33.3(1)	75.0(6)	0.543**
No	26.3(21)	80.0(4)		20.0(4)	25.0(5)	41.7(5)	31.8(7)	66.7(2)	25.0(2)	
**Non- informed consent interventions (medication administration, episiotomies, caesarean sections, etc.)**										
Yes	58.8(47)	60.0(3)	1.000**	70.0(14)	75.0(15)	41.7(5)	31.8(7)	66.7(2)	75.0(6)	0.028**
No	41.3(33)	40.0(2)		30.0(6)	25.0(5)	58.3(7)	68.2(15)	33.3(1)	25.0(2)	
**Lack of time devoted to women's care**										
Yes	61.3(49)	40.0(2)	0.646**	60.0(12)	65.0(13)	66.7(8)	45.5(10)	66.7(2)	87.5(7)	0.433**
No	38.8(31)	60.0(3)		40.0(8)	35.0(7)	33.3(4)	54.5(12)	33.3(1)	12.5(1)	
**Lack of privacy for the woman**										
Yes	83.8(67)	80.0(4)	1.000**	85.0(17)	70.0(14)	83.3(10)	90.9(20)	66.7(2)	100(8)	0.314**
No	16.3(13)	20.0(1)		15.0(3)	30.0(6)	16.7(2)	9.1(2)	33.3(1)	-	
**Women in labour being told they were endangering the life or health of their baby**										
Yes	11.3(9)	-	1.000**	20.0(4)	15.0(3)	-	-	-	25.0(2)	0.087**
No	88.8(71)	100(5)		80.0(16)	85.0(17)	100(12)	100(22)	100(3)	75.0(6)	
**Physical violence such as: repeated vaginal examinations, aggressive physical contact, interventions without anaesthesia, etc.**										
Yes	56.3(45)	40.0(2)	0.652**	60.0(12)	65.0(13)	41.7(5)	50.0(11)	33.3(1)	62.5(5)	0.743**
No	43.8(35)	60.0(3)		40.0(8)	35.0(7)	58.3(7)	50.0(11)	66.7(2)	37.5(3)	
**Kristeller manoeuvre to accelerate labour**										
Yes	47.5(38)	20.0(1)	0.368**	60.0(12)	60.0(12)	33.3(4)	22.7(5)	33.3(1)	62.5(5)	0.784**
No	52.5(42)	80.0(4)		40.0(8)	40.0(8)	66.7(8)	77.3(17)	66.7(2)	37.5(2)	
**Women's requests during childbirth being ignored**										
Yes	30.0(24)	60.0(3)	0.320**	35.0(7)	30.0(6)	25.0(3)	31.8(7)	-	50.0(4)	0.066**
No	70.0(56)	40.0(2)		65.0(13)	70.0(14)	75.0(9)	68.2(15)	100(3)	50.0(4)	
**Disclosure of private information without consent**										
Yes	10.0(8)	-	1.000**	-	20.0(4)	8.3(1)	4.5(1)	-	25.0(2)	0.126**
No	90.0(72)	100(5)		100(20)	80.0(16)	91.7(11)	95.5(21)	100(3)	75.0(6)	
**Shouting at or infantilizing women in labour**										
Yes	35.0(28)	-	0.166**	30.0(6)	55.0(11)	25.0(3)	18.2(4)	33.3(1)	37.5(3)	0.203**
No	65.0(52)	100(5)		70.0(14)	45.0(9)	75.0(9)	81.8(18)	66.7(2)	62.5(5)	
**Women were denied treatment they wanted or encouraged to accept interventions they did not want**										
Yes	28.7(23)	-	0.317**	25.0(5)	50.0(10)	8.3(1)	13.6(3)	-	50.0(4)	0.029**
No	71.3(57)	100(5)		75.0(15)	50.0(10)	91.7(11)	86.4(19)	100(3)	50.0(4)	
**What did you feel when witnessing these behaviours?**										
**Cold sweats**										

Yes	12.5(10)	-	1.000**	5.0(1)	10.0(2)	16.7(2)	13.6(3)	-	25.0(2)	0.681**
No	87.5(70)	100(5)		95.0(19)	90.0(18)	83.3(10)	86.4(19)	100(3)	75.0(6)	
**Tachycardia**										
Yes	28.7(23)	-	0.317**	25.0(5)	25.0(5)	16.7(2)	27.3(6)	33.3(1)	50.0(4)	0.705**
No	71.3(57)	100(5)		75.0(15)	75.0(15)	83.3(10)	72.7(16)	66.7(2)	50.0(4)	
**Difficulty concentrating**										
Yes	16.3(13)	20.0(1)	1.000**	10.0(2)	25.0(5)	16.7(2)	13.6(3)	-	25.0(2)	0.768**
No	83.8(67)	80.0(4)		90.0(18)	75.0(15)	83.3(10)	86.4(19)	100(3)	75.0(6)	
**Anxiety**										
Yes	58.8(47)	20.0(1)	0.078**	45.0(9)	45.0(9)	33.3(4)	45.5(10)	66.7(2)	37.5(3)	0.939**
No	41.3(33)	80.0(4)		55.0(11)	55.0(11)	66.7(8)	54.5(12)	33.3(1)	62.5(5)	
**Trouble sleeping**										
Yes	11.3(9)	-	1.000**	10.0(2)	10.0(2)	25.0(3)	4.5(1)	-	12.5(1)	0.564**
No	88.8(71)	100(5)		90.0(18)	90.0(18)	75.0(9)	95.5(21)	100(3)	87.5(7)	
**Agitation**										
Yes	40.0(32)	20.0(1)	0.644**	60.0(12)	50.0(10)	25.0(3)	18.2(4)	33.3(1)	38.5(3)	0.066**
No	60.0(48)	80.0(4)		40.0(8)	50.0(10)	75.0(9)	81.8(18)	66.7(2)	62.5(5)	
**Hypervigilance**										
Yes	50.0(40)	60.0(3)	1.000**	50.0(9)	40.0(8)	50.0(6)	63.6(14)	33.3(1)	50.0(4)	0.750**
No	50.0(40)	40.0(2)		50.0(9)	60.0(12)	50.0(6)	36.4(8)	66.7(2)	50.0(4)	
**None of the above**										
Yes	5.0(4)	95.0(76)	1.000**	5.0(1)	-	-	9.1(2)	33.3(1)	-	0.213**
No	-	100(5)		95.0(19)	100(20)	100(12)	90.9(20)	66.7(2)	100(8)	
**Have you considered dropping out of school (temporarily) due to situations experienced during your internship?**										
Yes	21.7(17)	-	0.577**	5.0(1)	25.0(5)	25.0(3)	18.2(4)	-	50.0(4)	0.124**
No	78.8(63)	100(5)		95.0(19)	75.0(15)	75.0(9)	81.8(18)	100(3)	50.0(4)	

Note: % = Percentage; (n) = Frequency; p = p-value; *Chi Square; **Fisher
